# Unintentional response priming from verbal action–effect instructions

**DOI:** 10.1007/s00426-022-01664-0

**Published:** 2022-04-02

**Authors:** Yevhen Damanskyy, Torsten Martiny-Huenger, Elizabeth J. Parks-Stamm

**Affiliations:** 1 UiT The Arctic University of Norway, Tromsø, Norway; 2 University of Southern Maine, Portland, Maine, USA

## Abstract

Action–effect learning is based on a theoretical concept that actions are associated with their perceivable consequences through bidirectional associations. Past research has mostly investigated how these bidirectional associations are formed through actual behavior and perception of the consequences. The present research expands this idea by investigating how verbally formulated action–effect instructions contribute to action–effect learning. In two online experiments (Exp. 1, *N* = 41, student sample; Exp. 2, *N* = 349, non-student sample), participants memorized a specific action–effect instruction before completing a speeded categorization task. We assessed the consequences of the instructions by presenting the instructed effect as an irrelevant stimulus in the classification task and compared response errors and response times for instruction-compatible and instruction-incompatible responses. Overall, we found evidence that verbal action–effect instructions led to associations between an action and perception (effect) that are automatically activated upon encountering the previously verbally presented effect. In addition, we discuss preliminary evidence suggesting that the order of the action–effect components plays a role; only instructions in a perception–action order showed the expected effect. The present research contributes evidence to the idea that action–effect learning is not exclusively related to actual behavior but also achievable through verbally formulated instructions, thereby providing a flexible learning mechanism that does not rely on specific actual experiences.

Many of our daily activities are aimed at achieving specific desired outcomes. However, how are specific actions selected to produce a desired outcome? The concept of action–effect learning, based on the principles of ideomotor theory, provides a basic idea for how intended outcomes can control our actions. The general idea is that specific actions trigger perceivable changes in one’s surroundings (i.e., effects), and the temporal proximity of these events results in the formation of associative links between actions and their perceivable consequences (action–effect associations, e.g., Elsner & Hommel, 2001). Because of these associative links, thinking about the effect will activate the linked behavior that previously produced the effect.

Empirical testing of the action–effect concept typically involves two stages (Elsner & Hommel, 2001; Greenwald, 1970). In the first stage (*learning phase*), participants experience the co-occurrence of specific actions and their effects (action–effect contingencies). The second stage (*test phase*) tests whether associations have been formed. In line with the assumption that such associations are bidirectional, exposing participants to previously encountered effects has been found to facilitate the respective associated actions (e.g., Elsner & Hommel, 2001; Paulus et al., 2011; Pfister, 2019; Shin et al., 2010). In the present research, we tested the idea that the acquisition of action–effect associations is not limited to actual behavior but can be acquired through verbal instructions.

## Verbally induced action control

In this section we summarize two research areas (i.e., implementation intentions and instruction implementation) that provide evidence that verbal information can influence subsequent action, potentially mediated by stimulus–response learning. Based on this evidence, we will then argue that verbal information about an action and an effect might also lead to action–effect learning.

The theory of implementation intentions suggests that behavior can be strategically controlled by forming a verbal plan in an *if–then* format (Gollwitzer, 1999). According to this theory, if–then planning creates direct perception–action links between the anticipated situation (critical cue) and the intended behavior (action). For instance, after forming the plan “If I pass a supermarket, then I will buy fruit,” the situation (supermarket) serves as a critical cue that triggers the planned action (buying fruit). Empirical laboratory tests of this idea are similar to the previously described action–effect learning procedure, except that during the learning phase, participants form specific verbal if–then action plans instead of actually enacting responses. In one example of such a test (Cohen et al., 2008, Exp. 2), participants memorized “If I hear the low tone on the left side, then I will press the right button especially fast.” In the test phase, participants were asked to perform a two-alternative forced-choice task (i.e., if the tone was high, they pressed the left button; if the tone was low, they pressed the right button). The results showed a response/verbal plan compatibility effect: required responses to the critical stimulus (if-part) were facilitated if they overlapped with the responses specified in the then-part of the plan. These and other similar results demonstrate that verbal (stimulus–response) planning influences subsequent behavioral responses (Cohen et al., 2008; Martiny-Huenger et al., 2017; Miles & Proctor, 2008).

Research from an instruction-based perspective provides additional evidence that instructions in the form of stimulus–response mappings can influence performance. The basic design of this type of research also involves a learning phase (verbal instructions) and a test phase. However, in many studies, the test phase is split into a *diagnostic task* and an *inducer task* (e.g., Liefooghe et al., 2012)*.* The given instructions are relevant for the inducer task but irrelevant for the diagnostic task. For instance, the instructions for the inducer task might read, “if you see ‘cat’, press left; if you see ‘dog’, press right.” However, before completing the inducer task, a preceding diagnostic task is introduced that shares both the stimuli (i.e., words ‘cat’ and ‘dog’) and responses (left/right button press) with the inducer task, but has different task instructions (e.g., to press the right or left button if the words are italicized or upright, respectively). Using this design, studies have demonstrated the presence of an instruction-based compatibility effect in the diagnostic task when the required response and the stimulus match the instructions given for the inducer task (e.g., when “cat” was italicized and required the left key response; for a review see Brass et al., 2017).

One of the fundamental differences between implementation intentions and instruction implementation research is that critical if–then sentences in implementation intention research are strongly highlighted and repeated as a central, important sentence to encode and remember (reviewed by Gollwitzer & Sheeran, 2006). Instruction-based research does not include such emphasis on a single sentence. The critical “if–then” instructions are just a part of the typical task instructions (Liefooghe & De Houwer, 2018; Liefooghe et al., 2012). Another central difference in these approaches is in the delay between reading the verbal plans/instructions and tests of their effects. If–then plans’ effects are tested minutes (in laboratory settings, e.g., Cohen et al., 2008) or even days or weeks later (e.g., Conner & Higgins, 2010; Papies et al., 2009). The effects of “instructions” are tested only seconds later (Brass et al., 2017). These time differences are relevant to the present research, and we will continue to discuss them later. In general, however, the two approaches share many similarities. For example, the verbal information in both cases typically includes a stimulus–response contingency. Both imply that verbally presented stimulus–response (perception–action) contingencies influence subsequent behavior.

## Verbal instructions within the action–effect paradigm

Theeuwes et. al. (2015) used a similar learning-test design in the context of action–effect learning. In three experiments, the authors provided instructions in an action–effect format in a learning phase and tested whether presenting the “effect” in a subsequent test phase would trigger the associated action. An example of an action–effect instruction from this research was: “If you press left, ‘P’ appears.” These instructions made sense to the participants as there was a part of the test phase in which participants produced the letter ‘P’ by pressing the left key (similar to the previously described inducer task in stimulus–response instruction-based research). Importantly, for testing action–effect learning, the letter ‘P’ also appeared as a target for a classification task (related to whether the letters were presented upright or italicized in the diagnostic task). The left and right key presses in the classification task established compatible and incompatible response trials with the action–effect instructions. The authors found that compatible responses (e.g., having to press the left key for the upright/italicized ‘P’) were facilitated compared to incompatible responses (e.g., having to press the right key in response to the upright/italicized ‘P’). Consequently, Theeuwes et. al. (2015) provide evidence that instructions that link an action to an effect can influence performance in an immediately followed (separated only by a few seconds) ostensibly irrelevant task.

## The present experiments

In the present research, we tested whether verbal action–effect instructions lead to associations between an action and an effect that are automatically activated upon perceiving the effect even if instructions and test are separated by more than a few seconds. We asked participants to memorize a specific verbal instruction that contained information about an action–effect relation (“To make the screen blue, I have to press the left key”). Afterward, participants performed a vowel-consonant categorization task. Although the task was unrelated to the action–effect instructions, responses in the categorization task overlapped with the responses specified in the action–effect sentence (i.e., left/right key). Importantly, on some trials, the screen background color turned blue (i.e., effect). This aspect was irrelevant for the categorization task and participants were instructed to ignore it. However, the presented blue background visually primed the effect from the action–effect instructions. We hypothesized that the priming of the effect would result in facilitated action–effect-compatible responses (i.e., categorization responses that align with the action–effect instructions) and/or in impaired incompatible responses (i.e., categorization responses that are different from those specified in the action–effect instructions).

While conceptually related to Theeuwes et. al. (2015), our present studies go beyond their evidence that action–effect instructions influence subsequent actions. We separated the processing of the instructions from the performance in the diagnostic task. To do this, we presented one action–effect instruction at the beginning of the experiment instead of continuously updating the instructions every 4, 6, or 16 trials. Thus, whereas Theeuwes et al. observed effects of instructions that participants read a few seconds earlier, we tested the effects of a single action–effect instruction presented to the participants a few minutes earlier (before reading other information like the categorization task instructions). Second, in the case of Theeuwes et. al. (2015), participants continuously performed inducer-task trials in the test phase, where the action–effect instructions were relevant after every 4, 6, or 16 trials. In the present work, participants were also told that the verbal action–effect instructions would be relevant at some point during the experiment. However, this information served only as a cover story and the participants never actually had to implement the instructions.

In sum, the effects of instructions on subsequent responses in Theeuwes et. al. (2015) were observed with instructions processed only seconds prior to testing their effects and in a context, where the participants were aware that the instructions were relevant just a few seconds later. In contrast, we tested effects with a longer time interval and in a context, where the instructions never had to be implemented and thus there were no explicit reminders of the action–effect instructions during the test phase. We conducted two online experiments. In the first experiment, we tested verbal action–effect instructions in an *effect*–*action* order. The central focus of the second experiment was to provide a direct replication of Experiment 1 with an increased sample size. In addition, we added an exploratory part in which we reversed the order of the instructions (*action*–*effect* order).

## Experiment 1

Participants memorized the action–effect instructions “To make the screen blue, I have to press the [left/right] key”. They then received additional instructions on how to perform the subsequent categorization task (press left/right for vowels/consonants). During this categorization task, the effect from the action–effect sentence (i.e., the blue screen background) was presented on a fourth of the trials. We hypothesized that perceiving the effect from the instructions should activate the verbally associated action and thus facilitate compatible responses (e.g., for blue-left instructions, perceiving blue and the left key is the required response) and/or interfere with incompatible responses (e.g., for blue-left instructions, perceiving blue and the right key is the required response).

In contrast to typical action–effect learning, where the action comes first, we presented the instructions in an effect–action format. This decision was driven by if–then planning research, where verbal information is given in a perception (if-part)–action (then part) order. Furthermore, in typical action–effect learning (and testing), a bi-directional link is required for an effect to trigger an associated response. As bi-directionality is an additional assumption that was not the central focus of our experiment, we decided to formulate the action–effect instructions in an effect–action format to align it with the to-be-encountered order in the test phase (i.e., perceiving the effect and executing the associated action; see “Experiment 2” for more information on the action–effect instruction order).

### Method

#### Participants

A total of 43 Norwegian-speaking adults participated in the study. Following data cleaning described in “Data analysis and data preparation approach” section below, the analyzed sample included the data of 41 participants (20 females, 20 males, and one missing gender response). The ages ranged from 19 to 51 (*M* = 24.14, SD = 5.04). The participants were compensated by participating in a drawing for one of two gift cards for a local shopping mall with a value of 500 NOK each. The study was approved by the local ethics committee, and all participants provided informed consent.

#### Design

Our design included two within-participant factors: *required response* (left key vs. right key) and *effect prime* (present vs. absent), and one between-participant factor: *instructed response* (press left key vs. press right key). *Required response* specified what response was required from participants in a given trial according to the categorization task instructions. *Effect prime* specified whether the blue screen was present (critical) or absent (neutral) in a given trial. *Instructed response* was a between-participant factor indicating the instructed action in the action–effect sentence (left key vs. right key; “To make the screen blue, I will press the left/right key”). Key assignment to vowel/consonant was counterbalanced between participants.

#### Procedure

The design and the procedure of this experiment originates from an unpublished experiment in a laboratory setting with various adjustments (see Appendix 1). The present experiment was conducted online and was programmed using PsychoPy v. 2020.1.3 and uploaded to the Pavlovia server (Pavlovia, 2021; Peirce et al., 2019). Each participant received a link to the experiment allowing them to open it in the browser of their choice. Participants were required to use a physical keyboard.

##### Learning phase

Participants were presented with an action–effect sentence: e.g., “*To make the screen blue, I will press the left key*” (in Norwegian: “Å gjøre skjermfargen blå, skal jeg trykke på venstretasten”). We presented an example of the critical stimulus (the color blue to be used in the study) prior to the action–effect instructions and told the participants that this would be the color that is referred to later in the instructions. To consolidate the instruction in memory, the participants were told to repeat the action–effect sentence silently to themselves a few times. We informed participants that this instruction would become relevant in a later task. Participants then received instructions for the test phase.

##### Test phase

The presented stimulus was either a vowel (A, Ø, or E) or a consonant (K, M, or T), and each appeared an equal number of times in random order. During this part, the participants judged whether a presented stimulus was a vowel by pressing the left key (A) or a consonant by pressing the right key (L). Along with each presented letter, the background color was either blue (effect prime present; 25% of the trials) or gray (effect prime absent; 75% of the trials). All stimulus and response combinations were equally distributed between the effect-present and effect-absent trials. We implemented a short response deadline. If a response was incorrect or longer than 1500 ms, an error feedback message was displayed for 1500 ms. Participants performed eight practice trials and 96 testing trials. The practice trials did not include any critical trials (i.e., the background was always gray).

#### Data analysis and data preparation approach

We used the R software package to prepare and analyze the data (R core Team, 2021). Response errors and reaction times were analyzed with a mixed ANOVA (*stats* package). Confidence intervals adjusted for the within-participant design were calculated by using *Rmisc* package (Hope, 2013). In addition, the reaction time variable was log-transformed (Judd et al., 1995). Responses other than A and L were removed prior to analyses (5.01% responses). No participant made “other” responses more than 50% of the time. Visual inspection of the data indicated one participant made an excessive number of fast responses. Therefore, we applied a criterion used in other online response-time studies (Greenwald et al., 2003; remove participant data with more than 10% responses faster than 300 ms). This resulted in the removal of the data from one participant. The boxplot method (Tukey, 1977) applied to mean error responses identified one participant as an extreme outlier (± 3 times the interquartile range) with a mean error rate of 20% (compared to the full sample’s mean error rate of 5.3%), so the data of this participant was also removed resulting in an analyzed sample size of 41 participants.

Individual trials were removed when the response deadline of 1500 ms was missed (0.51%). Prior to the response time analysis, we removed all error responses (5.3%). No responses were faster than 150 ms. We further removed trials with response times beyond the mean ± 3 times the standard deviation calculated by participant and within-participant conditions (1.07%).

## Results and discussion

### Response errors

All results of the ANOVA analysis with response errors as the dependent variable are presented in Table 1. In the following, we focus only on the hypothesis-relevant effects. The expected three-way interaction effect between required response, effect prime, and instructed response was marginally significant *F*(1, 39) = 3.50, *p* = 0.069, *η*_p_^2^ = 0.08. To explore this interaction effect, we analyzed response errors for prime present (critical) and prime absent (control) trials separately. For trials with the prime present, we found a significant two-way interaction effect between required response and instructed response*, F*(1, 39) = 6.61, *p* = 0.014, *η*_p_^2^ = 0.15. In contrast, for the control trials with the prime absent, the interaction effect was not significant, *F*(1, 39) = 0.62, *p* = 0.434, *η*_p_^2^ = 0.02.

**Fig. 1:**
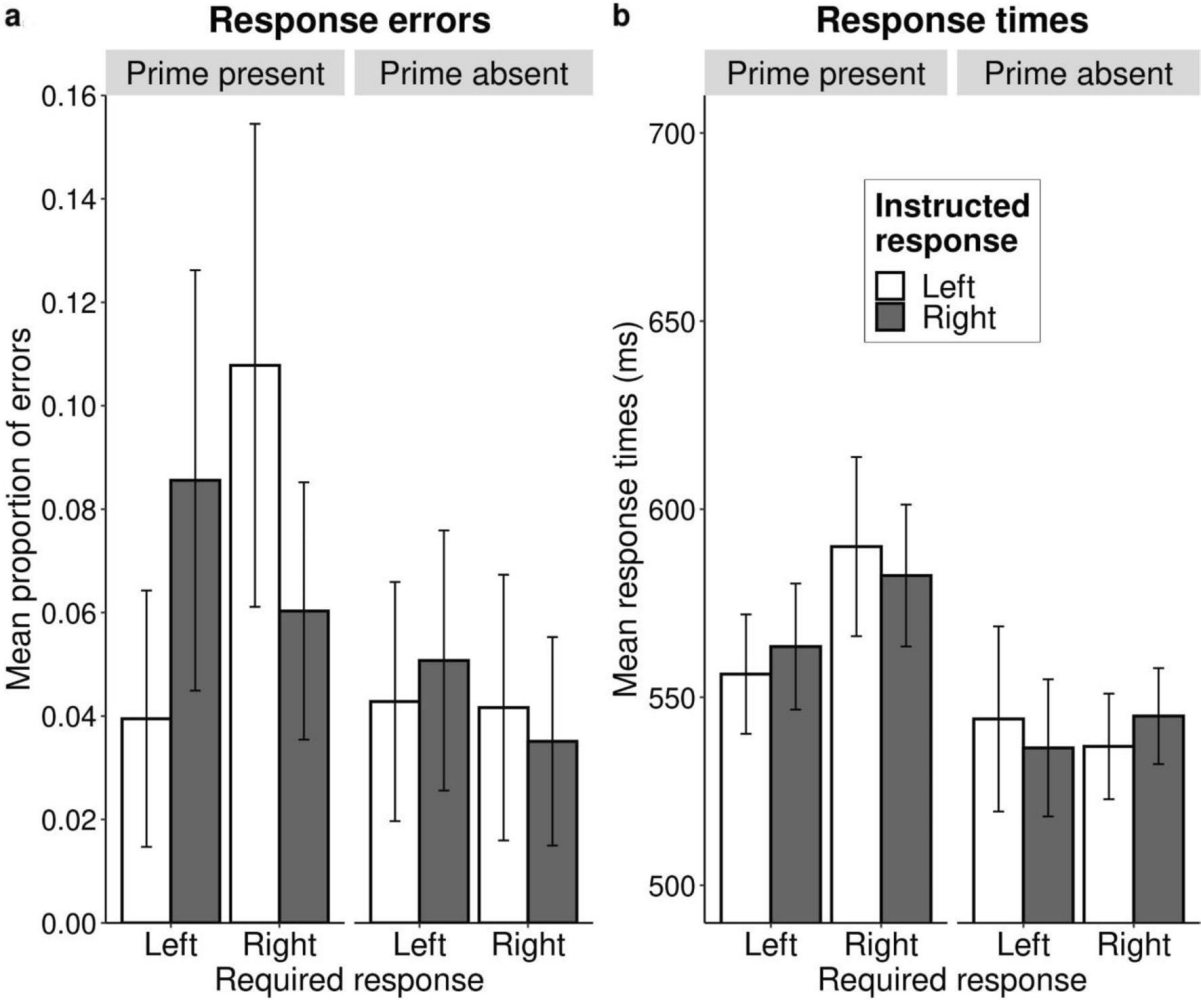
Mean response errors (**a**) and reaction time (**b**) as a function of required response, effect prime and instructed response. Bars represent descriptive means with the confidence intervals adjusted for the within-participant design according to the method of Morey-Cousineau (2008). The left pane **a** represents mean proportion of errors and the right pane **b** mean reaction times. *Required response* specifies what response was required from participants in a given trial according to the categorization task instructions. *Effect prime* specifies whether the blue screen was present (critical) or absent (neutral) in a given trial. *Instructed response* indicates the instructed action in the action–effect sentence (“To make the screen blue, I will press the left/right key”)

Despite the marginally significant result, the response error analysis showed that the pattern of results is in line with our predictions (see Fig. 1a). Presenting the action effect in a trial that required an incompatible response to the action–effect instructions (i.e., the action–effect instructions involved the right key and the required response was left or the action–effect instructions involved the left key and the required response was right) resulted in more errors than when the required response was compatible with the action–effect instructions (Fig. 1a, left pane). These differences were not observed in the control trials with the effect prime absent (Fig. 1a, right pane).

### Reaction time

All results of the ANOVA analysis with reaction time as the dependent variable are presented in Table 2. The analysis of reaction times revealed a main effect of prime *F*(1, 39) = 22.07 *p* =  < 0.001, *η*_p_^2^ = 0.36, indicating that participants responded more slowly on critical trials than neutral trials. The three-way interaction effect between required response, effect prime and instructed response was marginally significant *F*(1, 39) = 3.17, *p* = 0.083, *η*_p_^2^ = 0.08 (see Fig. 1b). We evaluated the descriptive pattern separately for the prime present and prime absent trials, to test whether the response-error pattern described in the previous section is further substantiated by a similar pattern in response times or whether it can instead be explained by a speed–accuracy trade-off (i.e., an effect in the opposite direction of the response errors). For trials with the effect prime present, the two-way interaction effect between required response and instructed response was not significant *F*(1, 39) = 0.71, *p* = 0.406, *η*_p_^2^ = 0.02. Similarly there was no significant two-way interaction effect for trials with the effect prime absent *F*(1, 39) = 1.01, *p* = 0.321, *η*_p_^2^ = 0.03. In sum, the response-time pattern (see Fig. 1b) indicates that the response-error pattern is not compromised by a speed–accuracy trade-off.

## Experiment 2

The first experiment provides initial evidence that the action–effect sentence influenced subsequent performance in response to the priming of the effect. However, we found this evidence only in response errors. Furthermore, although the separate analyses of the critical and control trials provided a clear picture, the overall three-way interaction effect was only marginally significant. A sensitivity analysis of the Experiment 1 data suggests that a mixed-design ANOVA with 41 participants across four within-conditions within two groups would be sensitive to an effect of *η*_p_^2^ = 0.21 with 80% power (*α* = 0.05). Given that the observed effect size was *η*_p_^2^ = 0.08, we conclude that the first experiment was underpowered. Therefore, we conducted a second experiment with the central focus of providing a higher powered exact replication of Experiment 1. If the result pattern found in Experiment 1 was due to chance, it is unlikely that a second, higher powered, independent replication would produce such a specific pattern again.

In addition to the central aim of replicating Experiment 1, we added an exploratory examination of an action–effect sentence formulated in a more typical *action*–*effect order* (“*I will press the left key to make the screen blue*”). This formulation of the instruction reflects the theoretical assumption of the action–effect principle that the associations resulting from action–effect learning are bidirectional; even if learning occurs in an action–then–effect order, encountering the effect first should trigger the response (e.g., Elsner & Hommel, 2001). Thus, Experiment 2 includes one part that is an exact replication of Experiment 1 with the effect (stimulus)–action (response) order format. Our hypotheses for this replication were the same as in Experiment 1: required responses that are incompatible (compatible) with the verbally linked, primed effect should be impaired (facilitated). The exploratory second part differed only in the order of the components (i.e., action [response]–effect [stimulus]). We had no specific hypotheses for this exploratory analysis. Whereas verbal if (stimulus)–then (response) planning research represents the order of presenting the verbal information as relevant, prior verbal action–effect studies have also found significant effects with an action (response)–effect (stimulus) order (Theeuwes et al., 2015). Whereas Experiment 1 participants consisted mainly of students from Norway (mean age 24.1), Experiment 2 participants were recruited from the general population of the United Kingdom (mean age 41.4).

## Method

### Participants

A total of 400 English speaking participants participated in the second experiment. Following data cleaning described in “Data analysis and data preparation approach” section below, the analyzed sample included the data of 173 participants in the replication study and 176 participants in the exploratory addition (199 females, 148 males, 2 missing responses). Their age ranged from 18 to 60 years (*M* = 41.9, SD = 12.1). Each participant was recruited by the recruiting agency Toluna (2021) and received a small monetary payment for taking part in the study. The study was approved by the local ethics committee, and all participants provided informed consent. A power analysis using the effect size from the first experiment showed that with *N* = 170 and *α* = 0.05, our mixed-design ANOVA had a power of *β* = 90% to detect the effect size reported in Exp. 1 (*η*_p_^2^ = 0.08).

### Design

The design was identical to the first experiment with two within-participant factors: *required response* (left vs. right)*, effect prime* (present vs. absent) and one between-participant factor *instructed response* (press left key vs. press right key). In addition, we introduced a separated condition: action–effect order. The additional condition allowed us to test both whether the effect–action order findings from Experiment 1 would replicate and whether we find an effect for the exploratory reversal of the component order (action–effect).

### Procedure

All materials were identical to the first experiment. In addition to the effect-order format (“*To make the screen blue, I will press the left key*”) presented in the first experiment and Part 1 of this second experiment, the additional instruction sentence was formulated in an action–effect format (e.g., “*I will press the left key to make the screen blue*”). As in the previous experiment, key assignment to vowel/consonant was counterbalanced between participants.

### Data analysis and data preparation approach

The data preparation procedure and outlier detection were identical to the first experiment. Prior to analysis, we removed the data of 3 participants who used different response keys than instructed more than 50% of the time (neither left “A” nor right “L”). Then we removed all individual responses that were neither ‘A’ or ‘L’ (1.5% of the total sample; accounted for by the same programming error as in Exp. 1). As in Experiment 1, we removed the data from 15 participants who made more than 10% of their responses below 300 ms (Greenwald et al., 2003). The response deadline of 1500 ms was missed in only 0.26% of trials. Using boxplot with interquartile range of ± 3 (Tukey, 1977), we removed the data of 26 participants with more than 22% response error. The full analyzed sample size was 349 participants.

Prior to the reaction time analysis, we removed all error responses (3.63%). We also excluded responses below 150 ms (i.e., fast guesses; 0.03% of the data) and trials with response times beyond the mean ± 3 times the standard deviation calculated by participant and within-participant conditions (1.15%).

## Results and discussion

### ***Effect***–***action order (replication)***

#### Response error

**Fig. 2:**
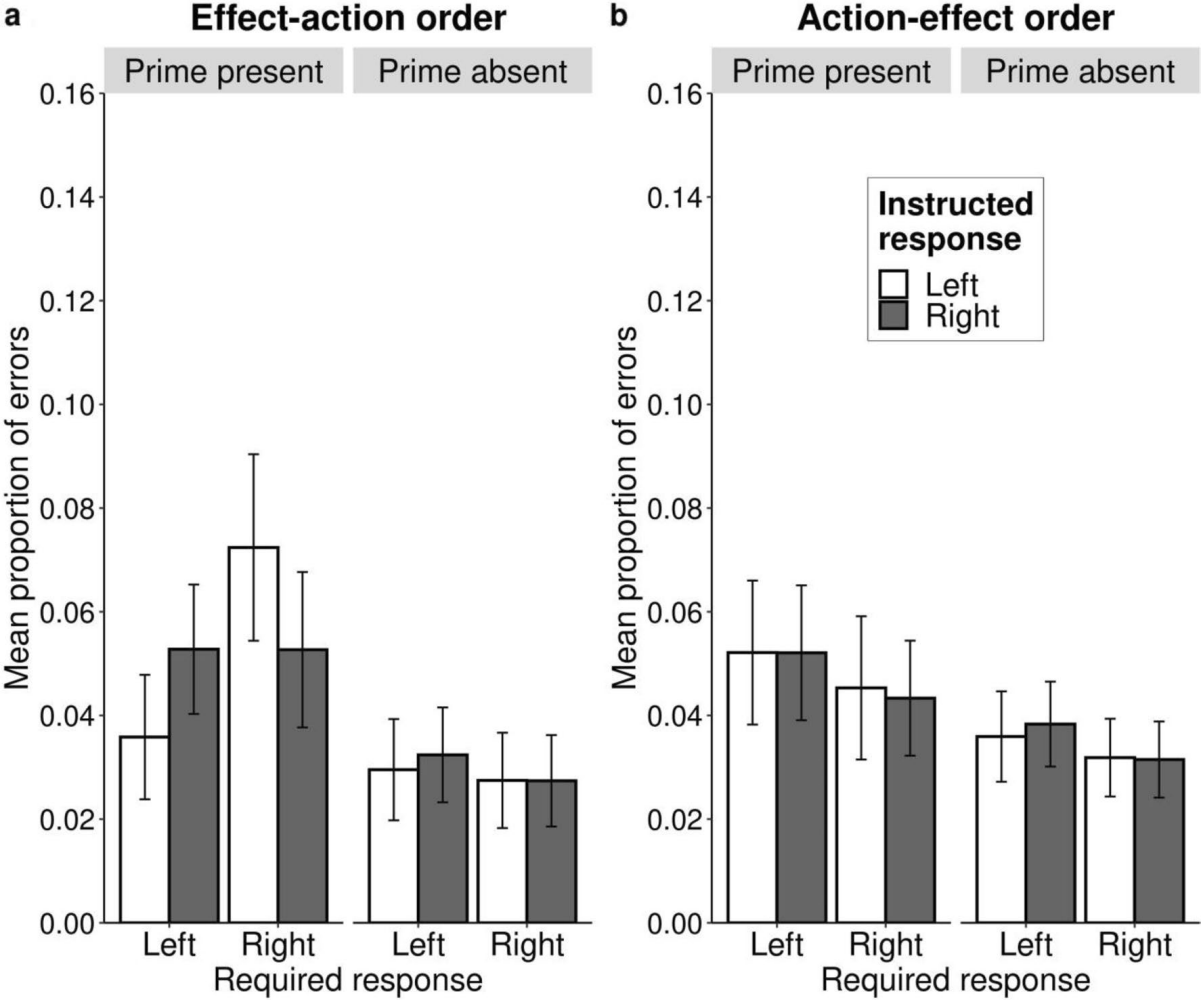
Mean response errors for effect–action sentence condition (**a**) and action–effect condition (**b**) as a function of required response, effect prime and instructed response (Replication of Experiment 1). Bars represent descriptive means with the confidence intervals adjusted for the within-participant design according to the method of Morey-Cousineau (2008). *Required response* specifies what response was required from participants in a given trial according to the categorization task instructions. *Effect prime* specifies whether the blue screen was present (critical) or absent (neutral) in a given trial. *Instructed response* indicates the instructed action in the action–effect sentence (“To make the screen blue, I will press the left/right key”)

All results of the ANOVA analysis with response errors as the dependent variable for the effect–action order are presented in Table 3. As in the first experiment, we focus only on the hypothesis-relevant effects. The expected three-way interaction effect between required response, effect prime, and instructed response is marginally significant *F*(1, 171) = 3.64, *p* = 0.058, *η*_p_^2^ = 0.02. As in Experiment 1, we evaluated the experimental effect further within the effect prime present (critical) and effect prime absent (control) trials separately. We found a significant two-way interaction effect in the effect prime present condition between required response and instructed response *F*(1, 171) = 5.41, *p* = 0.021, *η*_p_^2^ = 0.03. Whereas the same interaction effect was not significant within the effect prime absent trials *F*(1, 171) = 0.17, *p* = 0.680, *η*_p_^2^ < 0.01. In sum, in line with Experiment 1, when the effect prime was present, trials that required a response that was incompatible with the action–effect instructions resulted in more errors than responses that were compatible with the action–effect instructions (Fig. 2a, left pane). There was no such effect in the control trials with the effect prime absent (Fig. 2a, right pane). Thus, the results replicated the response error findings from Experiment 1.

#### Reaction time

**Fig. 3:**
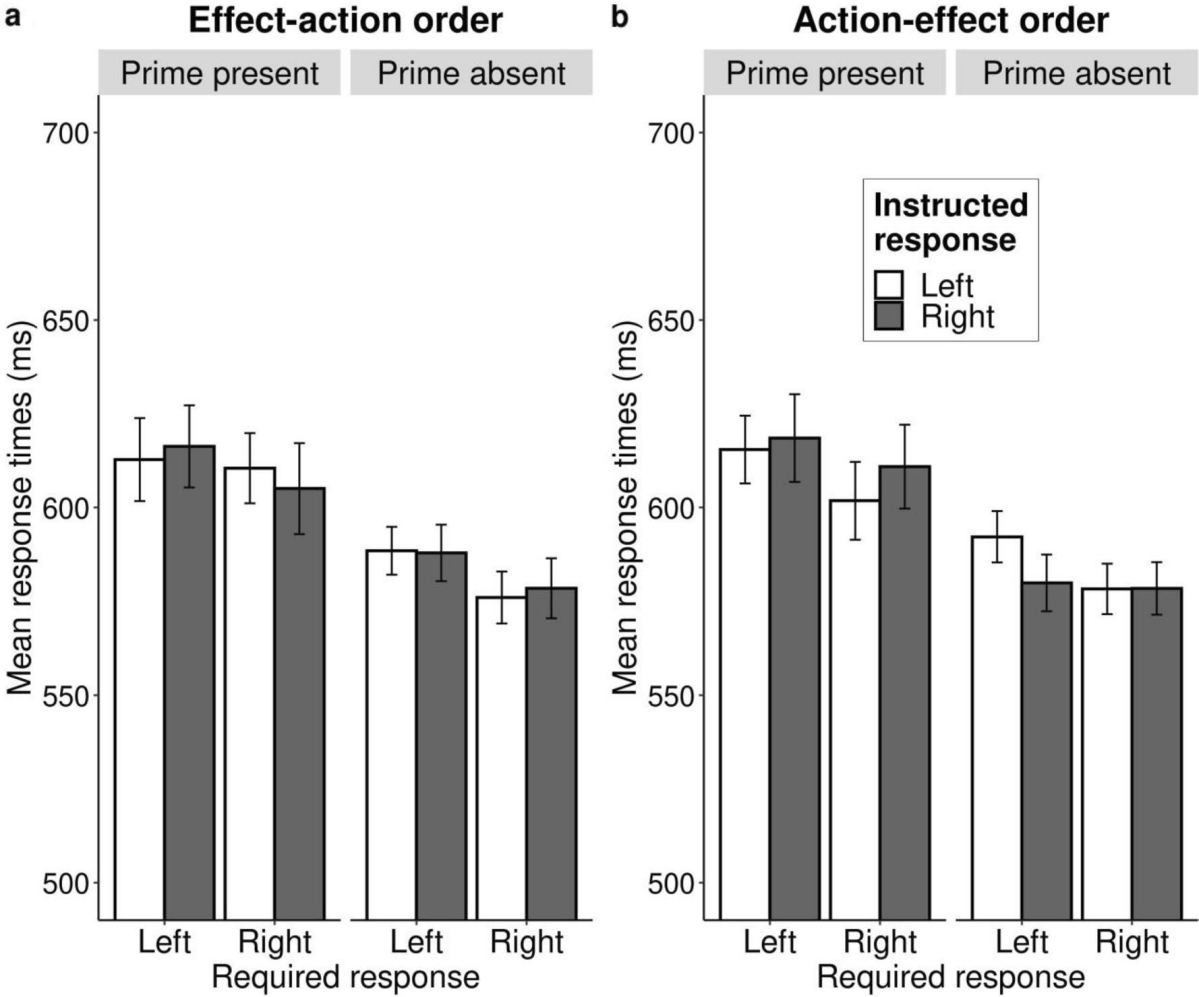
Mean response times for effect-action sentence condition (**a**) and action–sentence condition (**b**) as a function of required response, effect prime and instructed response. Bars represent descriptive means with the confidence intervals adjusted for the within-participant design according to the method of Morey-Cousineau (2008). *Required response* specifies what response was required from participants in a given trial according to the categorization task instructions. *Effect prime* specifies whether the blue screen was present (critical) or absent (neutral) in a given trial. *Instructed response* indicates the instructed action in the action–effect sentence (“To make the screen blue, I will press the left/right key”)

All results of the ANOVA analysis with reaction time as the dependent variable are presented in Table 4. The analysis revealed a significant main effect of prime *F*(1, 171) = 116.53 *p* < 0.001, *η*_p_^2^ < 0.41, indicating that participants responded slower on critical trials than on neutral trials. The three-way interaction effect between required response, effect prime, and instructed response was not significant *F*(1, 171) = 1.24, *p* = 0.268, *η*_p_^2^ < 0.01. As in the first experiment, we evaluated whether there was a speed–accuracy trade-off. The two-way interaction effect between required response and instructed response was not significant in trials with the effect prime present *F*(1, 171) = 0.52, *p* = 0.470, *η*_p_^2^ ≤ 0.01. The same analysis also did not show an effect in the control trials with the effect prime absent *F*(1, 171) = 0.18, *p* = 0.672, *η*_p_^2^ ≤ 0.01. As in the first experiment, these results indicate that the pattern of response errors were not affected by a speed–accuracy trade-off (Fig. 3a).

### ***Action***–***effect order***

#### Response errors

All results of the ANOVA analysis with response errors as the dependent variable for the action–effect order are presented in Table 5. The interaction effect between required response, effect prime, and instructed response was not significant *F*(1, 174) < 0.01, *p* = 0.960, *η*_p_^2^ < 0.01 (see Fig. 2b). Thus, we have no evidence that the instructions in the action–effect order influenced the responses.

#### Reaction time

Table 6 presents the results of the ANOVA analysis with reaction time as the dependent variable. Similar to the effect–action order, the analysis of the action–effect order showed a significant main effect of prime *F*(1, 174) = 79.65, *p* < 0.001, *η*_p_^2^ = 0.31, indicating that participants responded more slowly on critical trials than on neutral trials. We did not find a significant three-way interaction effect between required response, effect prime, and instructed response *F*(1, 174) = 0.30, *p* = 0.582, *η*_p_^2^ < 0.01, indicating that reaction times (Fig. 3b) were not influenced when the sentence was formulated in the action–effect order.

In sum, in Experiment 2 we replicated the effect observed in Experiment 1 by finding an effect of the action–effect instructions if the sentence was formulated in an effect (situation)–action (response) order. However, we found no effect of priming the effect when the instructions were formulated in an action (response)–effect (situation) order. It should be noted that the four-way interaction effect (required response × instructed response × effect prime × sentence-component order) did not reach significance, *F*(1, 345) = 2.16, *p* = 0.142, *η*_p_^2^ < 0.01. The decision to analyze the two parts of the experiment separately was guided by the aim to test whether the results of Experiment 1 were replicated. However, any conclusions based on the exploratory investigation of the order of the components can only be considered preliminary and should be interpreted with caution considering the non-significant four-way interaction effect.

## General discussion

In the present experiments we examined whether verbal action–effect instructions led to associations between perception (effect) and action that are automatically (i.e., unintentionally) activated upon encountering the effect. We tested this activation in behavioral responses in a speeded categorization task, where the effect was included as a task-irrelevant prime. Although some of the main findings were only marginally significant, the two experiments in combination revealed consistent evidence that the action–effect instructions (in an effect–action order) in combination with the effect prime influenced the accuracy of participants’ responses (with no evidence of a speed–accuracy trade-off). If the action effect prime was present, required responses that were incompatible with the instructed response showed more errors than when the responses were compatible with the previous action–effect instructions. Whereas Experiment 1 was underpowered, the replication in Experiment 2 (with four times the sample size) supported the results from Experiment 1. Why this increased sample size did not result in a clearer effect may be explained by the sample characteristics. There may have been increased random error variance from the significantly older, non-student sample in Experiment 2.

The result patterns could be interpreted as showing an interference effect in the effect-prime trials in which the previously verbally linked response was incompatible with the required response in the respective trial. However, facilitation from compatible response activation or interference from incompatible response activation can only be evaluated in comparison to an adequately similar control condition. The control condition in the present studies differed in terms of the critical priming factor (i.e., it did not include distracting sudden background-color changes). Assuming that the background color change negatively influenced responses in the prime/color-change trials, the absolute differences between critical and neutral trials are not comparable as we cannot estimate the size of that negative influence of the prime (i.e., prime main effect). Depending on the size of the prime/color-change induced interference, all combinations—only facilitation, facilitation and interference, or only interference—are possible. Investigating this would require a control condition that includes the same background-color change without including any (verbal) links of that color to a response. In such a condition, we could observe the consequences for responses induced merely by the sudden background-color change. Importantly, however, this limitation of not knowing whether facilitation, interference, or both caused the effect, does not reduce the informative value of the observed interaction effects, indicating that the verbal information systematically influenced the responses.

The absence of the hypothesized interaction effect in reaction times maybe explained by the response deadline. Response deadlines (i.e., forcing participants to emphasize speed over accuracy) typically leads to a reduced variability in response times and diminished power to detect reaction time effects (for a similar argument and findings in accuracy vs. reaction time measures, see Mekawi & Bresin, 2015). In sum, for the effect–action order formulation, we provide evidence that the verbally formulated perception–action relation—that was never directly experienced or executed—resulted in an association that was automatically reactivated upon perceiving the effect.

Our results align with previous research showing that imagining an effect while actually performing a response can lead to action–effect bindings (Cochrane & Milliken, 2019; Pfister et al., 2014). However, in the present research, participants did not previously experience the effect or response, but processed them merely as verbal action–effect instructions.

Eder and Dignath (2017) also showed action–effect learning from verbal instructions. However, in their test phase, participants experienced the previously instructed action–effect associations with each response. Therefore, it is not clear whether the observed effects were the direct effect of the instructions or some conflict between the instructions and the instruction-incompatible experiences. In our present experiments, participants never directly experienced the previously instructed action–effect contingency in the test phase. Thus, our study focused more narrowly on response priming from an instructed, verbal action–effect contingency. Finally, in contrast to the previously introduced research by Theeuwes et. al. (2015) in which instructions were likely to be kept in working memory (i.e., with responses given within a short interval after instructions were given), the present results indicate that the impact of instructions can have a longer lasting effect (beyond seconds and with processing other information in between), in line with the findings from implementation intention studies (Gollwitzer, 2014; Webb & Sheeran, 2008).

Martiny-Huenger et. al. () suggested a possible mechanism for this effect. According to their theoretical framework, verbal instructions that include a perceivable effect and executable action may work similarly to associative learning from direct processing and execution of the perception and action. This idea is based on theories of simulation and embodied cognition (Barsalou, 1999, 2010; Hesslow, 2012) and past findings that language comprehension of concrete concepts overlaps with sensorimotor areas activity of the brain (e.g., Arbib, 2008; Gallese & Lakoff, 2005; Pulvermüller & Fadiga, 2010). From this perspective, comprehension of verbal information activates the same sensorimotor brain areas that are involved during actual perception and behavior. Verbally processing a stimulus–response or action–effect contingency can thus result in the formation of specific associations between them—associations that are unintentionally activated upon encountering the perception (e.g., visual action effect) as suggested by our present experiments.

Studies on action–effect learning from direct experiences usually appear to form bi-directional links between action and effect, because the learning order (action, then effect) is reversed in the test phase (effect presentation, then action, e.g., Elsner & Hommel, 2001) However, the results from the second experiment indicated that the effect of the instruction sentence was only observed in the condition when the action–effect sentence was formulated in an effect–action direction (i.e., perception, then action: “*To make the screen blue, I will press the left key*”). In the action–effect order (action, then perception: “*I will press the left key to make the screen blue*”), the effect of the instructed sentence was not observed. These findings are not in line with the results of Theeuwes et. al. (2015), who only used the action–effect order and found effects of these instructions. If the present results prove to be robust in subsequent replications, a potential explanation could be in the differences of the procedure. Participants in the studies by Theeuwes et. al. (2015) were more likely to have kept the action–effect relation active in working memory. Thus, the order of the relation may be less important when the components are active in working memory. However, with the delay between processing the verbal instructions and executing the responses, whatever memory processes mediated the effects (e.g., associative learning), they may be sensitive to the order in which the components were processed before.

The statistically weak evidence for a difference between the two instruction component orders prohibits us from drawing strong conclusions about potential differences between the order of processing the action–effect components. However, our results are in line with a previous study by McCrea et. al. (2014), who investigated the consequences of differently formulated self-regulation instructions before doing a prospective memory task. Although the authors modeled instructions to fit different theoretical concepts, one of the instructions included a stimulus–response order that was similar to our effect–action order (“Whenever I see the red circle, then I will immediately press the spacebar”). The other two formulations included a response–stimulus order (e.g., “I will immediately press the spacebar when I see the red circle!”) similar to our action–effect order. Like our findings, only the stimulus–response order (i.e., perception–action) was effective in their study (McCrea et al., 2014). More anecdotally, in the initial publications of if–then planning research, the strategy was sometimes presented in a response–stimulus format (e.g., “I intend to do y when situation z is encountered”; Gollwitzer, 1993; Gollwitzer & Brandstätter, 1997**)**. However, at some point, this changed, and subsequent publications almost exclusively used the if (stimulus)–then (response) order (e.g., “When situation x arises, I will perform response y!”; Gollwitzer, 1999). This could have been the result of a mere refinement of the concept, or as a result of practical experience that the reversed order (response–situation) is less effective.

Why might the perception–then–action order be more effective than the action–then–perception order (at least in measures after a few seconds)? Disregarding the rich subjective experiences that we associate with language in general and discussing it from the perspective of simulation accounts of cognition alone might provide an interesting answer. As argued previously, repeating the presented instructions in the presented form may act as a placeholder for the actual experiences. From this actual-experience perspective and the fact that reading is sequential—the order of the components in the instructions results in differences in whether the perception (e.g., effect) is predictive of a response or not. In our effect–action order and McCrea et. al.’s (2014) stimulus–response order, the perception (effect/stimulus) is followed by the response; the perception part is thus predictive of the action part. During the test phase, the perception is there first (effect prime, blue screen) and the perception, therefore, biased actual responses in line with the prior learning. In contrast, in the action–effect order and McCrea et. al.’s (2014) response–stimulus order, the perception of the effect/stimulus was not predictive for the action, because in this case, the action preceded the perception. Thus, when the perception occurred in the test phase, it did not have any systematic predictive value and thus did not bias the subsequent responses.

Whereas the evidence we present for such an order effect in the present research is weak, it lines up with other prior evidence (e.g., McCrea et al., 2014; if–then planning research in general). Furthermore, where it conflicts with prior evidence (e.g., Theeuwes et al., 2015), it can easily be reconciled with differences in the procedures (i.e., instructions kept in working memory for a few seconds vs. effects that could not have been kept in working memory). More research is needed to support the reliability of a systematic difference between the component order. In addition to the new theoretical questions about action–perception learning raised by these findings, the present study contributes to the idea that language is intertwined with action control (Perlovsky & Sakai, 2014) and can be strategically used to control our behavior (Gollwitzer, 1999; Martiny-Huenger et al., 2017).

## Conclusions

In the present work, our findings showed that action–effect associations can be formed through verbal instructions. Although the perception–action relation presented as action–effect instructions was never executed by the participants before, it still had unintentional consequences when the perception component (effect) was encountered in the instruction-irrelevant classification task. We interpret these findings as evidence that verbal instruction can serve as a learning process in addition to learning from actual behavior. The complexity of human behavior would be hard to imagine if learning was limited to learning from actual behavior. The unrestricted combinatory potential of language allows us to learn relations that we have never actually experienced before in such a combination. Importantly, our present research suggests that such learning from language does not necessarily happen only at the declarative knowledge level (Anderson, 1982), but that encountering verbal perception–action contingencies might directly influence procedural knowledge.

## Data Availability

The data sets are available via the Open Science Framework (OSF): https://osf.io/w72mx/?view_only=85d511d72bd646138a00ef71f107abd7.

## Appendix 1

For transparency reasons, we want to disclose that prior to conducting the two online experiments presented in this manuscript, we conducted one more experiment in a laboratory setting (*N* = 50) aimed to test the same hypothesis (this first experiment included a pre-registration on Open Science Framework (https://osf.io/w2j53) of the hypothesis tested in the present manuscript. The results did not reveal any compatibility effects. We decided not to include this study in the main manuscript because of a technical issue that resulted in half of the Norwegian participants receiving instructions in English. There was no way to know which participants received the incorrect instructions. Thus, considering the importance of language in our design (the experimental manipulation is contained in a single sentence), it is hard to evaluate the meaningfulness of the experiment. In addition, there were significant procedural differences as we improved the procedure and simplified our approach: originally, responses were done with two joysticks by making left or right push movements (as compared to pressing a left or right key in the present experiments). In addition, we used several colors (red, green, yellow, blue) as the background colors. Practically, this resulted in a rather distracting background color change on each trial (as compared to the more stable grey background color with occasional switches to the critical blue color in the present experiments). Furthermore, participants performed 194 trials in the testing phase, which could have potentially diminished an experimental effect due to learning during the test-task execution (see Schmidt et al., 2016); the present studies contained only 96 trials. Finally, we realized that participants may imagine different variations of the color “blue” when reading the critical action–effect sentence. To establish a single color that would be more consistently imagined between participants and more consistent with what they would see in the test phase, we showed the critical color to participants in the present study (before they learned the action–effect instructions) and told them that this would be the color that later instructions would refer to.

## Appendix 2

See Tables 1, 2, 3, 4, 5 and 6.

**Table 1:** Anova results for experiment 1 (response errors)

Predictors	df	Sum of squares	*F*	*p*	*η*_p_^2^
Instructed response	1, 39	0.01	0.87	0.352	0.02
Required response	1, 39	< 0.01	0.45	0.506	0.01
Required response × instructed response	1, 39	0.03	7.63	0.009	0.16
Effect prime	1, 39	0.04	8.17	0.007	0.17
Effect prime × instructed response	1, 39	< 0.01	< 0.01	0.948	< 0.01
Required response × effect prime	1, 39	< 0.01	2.00	0.165	0.05
Required response × effect prime × instructed response	1, 39	0.02	3.50	0.069	0.08

**Table 2:** Anova results for experiment 1 (reaction times)

Predictors	df	Sum of squares	*F*	*p*	*η*_p_^2^
Instructed response	1	11,438	0.33	0.570	< 0.01
Required response	1	7236	3.50	0.069	0.08
Required response × instructed response	1	1	< 0.01	0.980	< 0.01
Effect prime	1	41,628	22.07	< 0.001	0.36
Effect prime × instructed response	1	1	< 0.01	0.979	< 0.01
Required response × effect prime	1	6634	8.93	0.005	0.19
Required response × effect prime × instructed response	1	2357	3.17	0.082	0.08

**Table 3:** Anova results for experiment 2 (effect–action sentence; response errors)

Predictors	df	Sum of squares	*F*	*p*	*η*_p_^2^
Instructed response	1	0.007	0.88	0.350	< 0.01
Required response	1	0.009	3.03	0.084	0.02
Required response × instructed response	1	0.017	5.48	0.020	0.03
Effect prime	1	0.101	29.09	< 0.001	0.15
Effect prime × instructed response	1	< 0.001	0.10	0.758	< 0.01
Required response × effect prime	1	0.020	6.07	0.015	0.03
Required response × effect prime × instructed response	1	0.012	3.64	0.058	0.02

**Table 4:** Anova results for experiment 2 (effect–action sentence; reaction time)

Predictors	df	Sum of squares	*F*	*p*	*η*_p_^2^
Instructed response	1	35,739	1.14	0.286	< 0.01
Required response	1	13,527	4.33	0.039	0.02
Required response × instructed response	1	375	0.12	0.729	< 0.01
Effect prime	1	138,723	116.53	< 0.001	0.41
Effect prime × instructed response	1	153	0.13	0.721	< 0.01
Required response × effect prime	1	757	0.61	0.437	< 0.01
Required response × effect prime × instructed response	1	1540	1.24	0.268	< 0.01

**Table 5:** Anova results for experiment 2 (action–effect sentence; response errors)

Predictors	df	Sum of squares	*F*	*p*	*η*_p_^2^
Instructed response	1	0.004	0.67	0.410	< 0.01
Required response	1	0.008	2.59	0.110	0.01
Required response × instructed response	1	< 0.001	0.08	0.780	< 0.01
Effect prime	1	0.033	15.90	< 0.001	0.08
Effect prime × instructed response	1	< 0.001	0.09	0.770	< 0.01
Required response × effect prime	1	< 0.001	0.09	0.760	< 0.01
Required response × effect prime × instructed response	1	< 0.001	< 0.01	0.960	< 0.01

**Table 6:** Anova results for experiment 2 (action–effect sentence; reaction time)

Predictors	df	Sum of squares	*F*	*p*	*η*_p_^2^
Instructed response	1	17,928	0.43	0.511	< 0.01
Required response	1	14,603	6.49	0.012	0.04
Required response × instructed response	1	3697	1.64	0.202	< 0.01
Effect prime	1	150,868	79.65	< 0.001	0.31
Effect prime × instructed response	1	6425	3.39	0.067	0.02
Required response × effect prime	1	381	0.26	0.609	< 0.01
Required response × effect prime × instructed response	1	443	0.30	0.582	< 0.01

## Code availability

The codes used to analyze the data are available via the Open Science Framework (OSF): https://osf.io/w72mx/?view_only=85d511d72bd646138a00ef71f107abd7.
